# Supplementary material for: TomoGRAF: An X-ray physics-driven generative radiance field framework for extremely sparse view CT reconstruction
Source: PLoS One. 2025 Aug 22;20(8):e0330463. doi: 10.1371/journal.pone.0330463 (PMC12373210; doi:10.1371/journal.pone.0330463)
Supplement: S1 Appendix — (DOCX) [file pone.0330463.s001.docx]

| I**NPUT**:  Ray start point: $\boldsymbol{x}_{\boldsymbol{0}}\boldsymbol{(}x_{0}\boldsymbol{,}y_{0}\boldsymbol{,}z_{0}\boldsymbol{)}$  Ray end point: $\boldsymbol{x}_{\boldsymbol{1}}\boldsymbol{(}x_{1}\boldsymbol{,}y_{1}\boldsymbol{,}z_{1}\boldsymbol{)}$  Grid Spacing: $\boldsymbol{\Delta x (}\Delta x, \Delta y, \Delta z\boldsymbol{)}$  Grid bounds $\boldsymbol{x}_{\boldsymbol{min}}\boldsymbol{(}x_{min}\boldsymbol{,}y_{min}\boldsymbol{,}z_{min}\boldsymbol{)}$**,** $\boldsymbol{x}_{\boldsymbol{max}}\boldsymbol{(}x_{max}\boldsymbol{,}y_{max}\boldsymbol{,}z_{max}\boldsymbol{)}$ |
| --- |
| **OUTPUT**:  Intersected pixels location and their corresponding path lengh. |
| **INITIALIZE:**  $dx = x_{1} - x_{0}$  $dy = y_{1} - y_{0}$  $dz = z_{1} - z_{0}$  // Ray length  $L = sqrt({dx}^{2}+ {dy}^{2} + {dz}^{2})$  // Unit vector components  $ux = \frac{dx}{L}$  $uy = \frac{dy}{L}$  $uz = \frac{dz}{L}$  // Entry and exit distances  $tx_{min}=\frac{\left( x_{min} - x_{0} \right)}{ux} if ux \neq0 else \pm\infty$  $tx_{max} = (\frac{\left( x_{max} - x_{0} \right)}{ux} if ux \neq0 else \pm\infty$  $ty_{min}=\frac{\left( y_{min} - y0 \right)}{uy}if uy \neq0 else \pm\infty$  $ty_{max}=\frac{\left( y_{max}- y_{0} \right)}{uy} if uy \neq0 else \pm\infty$  $tz_{min}=\frac{\left( z_{min} - z0 \right)}{uz} if uz \neq0 else \pm\infty$  $tz_{max}=\frac{\left( z_{max} - z0 \right)}{uz}if uz \neq0 else \pm\infty$  // Largest entry distance  $t_{entry} = max(tx_{min}, ty_{min}, tz_{min})$  // Smallest exit distance  $t_{exit} = max(tx_{max}, ty_{max}, tz_{max})$  IF t_entry > $t$_exit OR $t$_exit < 0:  // Ray misses the grid  RETURN empty list  Initialize voxel indices $(i, j, k)$ for the starting voxel  Compute first voxel intersections $(t_{x},t_{y}, t_{z})$  Set step ($\Delta t_{x}$, $\Delta t_{y}$, $\Delta t_{z}$) based on ray direction  Calculate  $\Delta t_{x}= \frac{\Delta x}{\left\vert ux \right\vert}$  $\Delta t_{y}= \frac{\Delta y}{\left\vert uy \right\vert}$  $\Delta t_{z}= \frac{\Delta z}{\left\vert uz \right\vert}$ |
| **MAIN LOOP:**  While t$t_{entry}$ ≤$t_{exit}$:  Identify the next boundary to cross:  IF$t_{x} \leq t_{y}$ AND $t_{x} \leq t_{z}$:  Advance to next voxel in x-direction  Update$t_{x} = t_{x} + \Delta t_{x}$  ELSE IF $t_{y}\leq t_{x}$ AND $t_{y}\leq t_{z}$:  Advance to next voxel in y-direction  Update $t_{y} = t_{y} + \Delta t_{y}$  ELSE:  Advance to next voxel in z-direction  Update $t_{z} = t_{z} + \Delta t_{z}$  Compute path length $\Delta t$for the current voxel  Record voxel index $(i, j, k)$ and path length $\Delta t$  END LOOP |
| **RETURN**:  List of intersected voxels with their respective path lengths |

**Appendix 1**: Siddon’s Ray Tracing algorithm pseudo code applied in TomoGRAF projection rendering module.
