# Supplementary material for: TomoGRAF: An X-ray physics-driven generative radiance field framework for extremely sparse view CT reconstruction
Source: PLoS One. 2025 Aug 22;20(8):e0330463. doi: 10.1371/journal.pone.0330463 (PMC12373210; doi:10.1371/journal.pone.0330463)
Supplement: Attachment — Submitted filename: Comments.docx [file pone.0330463.s002.docx]

The authors present TomoGRAF, an innovative approach leveraging ultra-sparse projections to achieve high-quality 3D CT volume reconstruction, marking a significant advancement in X-ray physics and CT imaging. This work introduces the first universal framework for image-guided radiotherapy and interventional radiology, offering substantial clinical utility. While the manuscript demonstrates considerable promise for publication in PLOS ONE, the authors should address the following points prior to final acceptance.

1. Most NeRF methods rely on 2D supervision, while this paper mentions using 3D CT as the supervision signal, but does not explicitly explain how to ensure the effectiveness of 3D supervision during training. How can 3D supervision improve fidelity? It is suggested to add relevant ablation experiments or analysis to demonstrate the specific impact of 3D supervision on the final reconstruction quality.
2. The paper claims to model X-ray attenuation, but it is unclear whether scattering, beam hardening, or noise were considered. These factors are crucial for real CT simulations.
3. The paper only mentions two data augmentation techniques - random flipping and rotation. Were other data augmentation methods employed, such as noise injection or varying SID/SAD, to improve generalization capability?
4. For the loss functions in Equation (11) and Equation (12), how were the parameters α and β determined? Were ablation experiments conducted to ensure they are optimal values? For the loss function in Equation (13), how were the parameters γ, δ, and ε determined? And how were θ and φ determined in the evaluation metrics?
5. In the experiments, why was the peak signal-to-noise ratio (PSNR) = 25 set as the stopping threshold? Why not choose a higher PSNR value?
6. During the experimental process, your model's baseline performance was established using a single AP view. To determine model performance, reconstructions were additionally performed with 1, 2, 5, and 10 views. The view angles were specified as follows: for 1-view reconstruction, the AP view was used as reference; for 2-view reconstruction, AP and lateral views were used for inference; for 5-view reconstruction, starting from the AP view, every 72° rotation was applied to cover the full 360°; for 10-view reconstruction, starting from the AP view, every 36° rotation was applied to cover the full 360°. However, in the Discussion section, the statement "Meanwhile, TomoGRAF, besides 1-view referencing, can leverage additional X-ray views at arbitrary angles" appears to lack strong experimental support, since the experimental procedure in this study clearly defined view angles with uniform angular intervals, which seems inconsistent with "arbitrary angles". We recommend either: (1) adding experiments to demonstrate the value of arbitrary angles (e.g., using only the AP view and its adjacent angles) for TomoGRAF during inference, or (2) rephrasing this statement to better align with the actual experimental conditions.
7. TomoGRAF requires fine-tuning during the inference phase. For specific fine-tuning, the trained prior model is optimized under the supervision of the patient's 2D sparse view projections to adapt to new patient anatomies. Does this mean that during the inference phase, fine-tuning is required for projection images from every angle used? If so, this would lead to increased inference time, and when more views are used for inference, the time would increase accordingly. The paper mentions that single-view reconstruction takes approximately 344 seconds, while each additional view roughly doubles the reconstruction time, which appears relatively slow for practical applications. Can the network be improved to enhance its computational efficiency?
8. In the quantitative analysis, this paper employed SSIM, PSNR, and RMSE metrics, but it did not thoroughly discuss in the qualitative analysis whether these metrics can reflect clinical diagnostic requirements. In clinical applications, the accuracy of CT images is crucial. Have you considered inviting clinicians to evaluate the generated predicted CT images and assess their feasibility?
9. The pseudocode of the Siddon algorithm (Appendix 1) serves as an important methodological supplement. Please further clarify its specific implementation details in TomoGRAF, such as how it integrates with the fully connected network - does the network directly learn attenuation coefficients, or is this achieved through post-processing?
