# Supplementary material for: TomoGRAF: An X-ray physics-driven generative radiance field framework for extremely sparse view CT reconstruction
Source: PLoS One. 2025 Aug 22;20(8):e0330463. doi: 10.1371/journal.pone.0330463 (PMC12373210; doi:10.1371/journal.pone.0330463)
Supplement: Attachment — Submitted filename: Response to Reviewers_Final.docx [file pone.0330463.s004.docx]

Dear Editor and Reviewers,

We thank you for your insightful comments, which helped us improve the manuscript. We responded to the comments individually as follows and revised the manuscript accordingly. We are happy to address any additional comments or critiques.

Sincerely

Authors.

1. Most NeRF methods rely on 2D supervision, while this paper mentions using 3D CT as the supervision signal but does not explicitly explain how to ensure the effectiveness of 3D supervision during training. How can 3D supervision improve fidelity? It is suggested to add relevant ablation experiments or analysis to demonstrate the specific impact of 3D supervision on the final reconstruction quality.

We thank the reviewer for highlighting the importance of analyzing the effect of 3D supervision. Compared to prior NeRF-based approaches such as MedNeRF, our TomoGRAF framework introduces two major enhancements to improve reconstruction fidelity:

1) a more physically accurate forward model using Siddon’s ray tracing, and
2) the incorporation of 3D supervision from paired CT volumes during training.

These two design choices work in tandem to enhance anatomical consistency, especially under ultra-sparse-view constraints. Since our model ultimately generates 3D volumes at inference time, incorporating 3D supervision during training is both intuitive and effective—it aligns the learning objective with the test-time task and enables the model to learn more robust and structured anatomical priors.

We agree that many existing NeRF-based medical imaging frameworks rely on 2D supervision primarily due to the unavailability of paired 3D data for objects viewed under natural lights, rather than by design choice. In contrast, TomoGRAF is trained on paired 2D–3D data, giving the unique advantage of X-ray imaging in visualizing complex 3D anatomical structures.

While we agree that an ablation study isolating the impact of 3D supervision would be informative, the current manuscript, which is already lengthy, includes extensive evaluations across various sparse-view configurations. To maintain focus and clarity, we limited the scope to demonstrating the feasibility and effectiveness of the proposed framework. A deeper exploration of supervision strategies and architectural contributions should belong to a separate future study.

2. The paper claims to model X-ray attenuation, but it is unclear whether scattering, beam hardening, or noise were considered. These factors are crucial for real CT simulations.

We thank the reviewer for raising this important point. Our current implementation of the forward model uses Siddon’s ray tracing algorithm, which simulates X-ray attenuation as line integrals through a voxelized volume. This approach assumes a monochromatic source and neglects scattering, beam hardening, and noise. While this simplified model is commonly used in simulation studies due to its computational efficiency and clarity, we agree that these effects are relevant to actual image quality. Future work will incorporate more realistic forward models that simulate polychromatic spectra, scatter, and noise characteristics in sparse reconstruction using actual projection X-ray images. That said, the focus of this study is to evaluate theoretical potential of TomoGRAF under ideal conditions.

3. The paper only mentions two data augmentation techniques - random flipping and rotation. Were other data augmentation methods employed, such as noise injection or varying SID/SAD, to improve generalization capability?

We thank the reviewer for this thoughtful question. In this work, we employed random flipping and rotation as basic data augmentation strategies. We did not apply noise injection or vary geometric parameters such as SID/SAD during training, for the following reasons:

1) SID/SAD Parameters: These values were extracted directly from each patient’s DICOM metadata and therefore naturally vary across the cohort, reflecting realistic acquisition conditions without the need for synthetic augmentation.
2) Noise Injection: Our training cohort includes over 1,000 patients, each contributing multiple view angles and patches for supervision, offering substantial variability in anatomy, pose, and appearance. Since our method is designed to learn a universal prior during training—rather than rely on patient-specific fine-tuning or enhance the robustness of a CNN inference model—we determined that additional noise injection was unnecessary.
3) Purpose of the Prior: Since the prior is trained to capture general anatomical structure across patients and is later fine-tuned to sparse-view test data, our focus was on extracting shared structural features rather than simulating acquisition-specific degradations (which are better handled during test-time optimization).

That said, we agree that investigating the effect of more aggressive augmentations (including noise) could be a direction for further robustifying the prior, especially when adapting TomoGRAF to real clinic which requires institution-specific fine tuning on the prior. The additional augmentation is unnecessary for the current study which is based on relatively large and diverse datasets.

4. For the loss functions in Equation (11) and Equation (12), how were the parameters α and β determined? Were ablation experiments conducted to ensure they are optimal values? For the loss function in Equation (13), how were the parameters γ, δ, and ε determined? And how were θ and φ determined in the evaluation metrics?

The parameters α, $\phi$, γ, δ, and ξ were not used in Equations (11-12). The parameters $\phi,$ α and $\boldsymbol{\xi}$ were mentioned and defined in Page 12 as “Overall, the generator $G$ takes x-ray source setup matrix $\boldsymbol{K}$, view direction (pose) $\boldsymbol{\xi}=(\theta,\phi)$ , 2D sampling pattern $v$, shape code $z_{sh}\in\mathbb{R}^{M_{\boldsymbol{s}}}$ and appearance code $z_{a}\in\mathbb{R}^{M_{\boldsymbol{a}}}$ as input” as highlighted in green in the manuscript, where $\phi$ is the elevation angel, and $\theta$ is the azimuthal angel of view position pose $\boldsymbol{\xi}$, and appearance code $z_{a}$ is a latent vector that encodes view-dependent features and is optimized by the model during training. The parameter $\delta$ were mentioned in Page 12 as “The output is the material density $\delta$ in the corresponding $\boldsymbol{x}$, where $\vartheta$ represents the network parameters and $z_{sh}\sim p_{sh}$ and $z_{a}\sim p_{a}$ with $p_{sh}$ and $p_{a}$ drawn from standard Gaussian distribution.” (highlighted in green in the manuscript), where $\delta$ represents the material density within the 3D CT volume which needs to be learnt by the trained model. The parameter $\gamma$ was defined in page 14 as “Where $\mathcal{L}_{x}$ and $\mathcal{L}_{\xi}$ represent the latent codes of $\boldsymbol{x}$ and $\boldsymbol{\xi}$, $M_{\boldsymbol{sh}}$ and $M_{\boldsymbol{a}}$ define the shape and appearance codes with $z_{sh}\in\mathbb{R}^{M_{\boldsymbol{sh}}}$ and $z_{a}\in\mathbb{R}^{M_{\boldsymbol{a}}}$, and $\gamma(\cdot)$ represents positional encoding.” (highlighted in green in the manuscript), where $\gamma(\cdot)$ represents positional encoding of the model. We did not use β in Equation (11-13) and throughout the manuscript.

Additionally, in Equations (11-13), hyperparameters $\lambda_{1}$, $\lambda_{2}{, \lambda}_{3},\lambda_{4}$and $\lambda_{5}$ are used to control the relative contributions of different loss terms (Equations 11–13) and metric components. These values were empirically chosen based on standard practices in the literature and preliminary experiments to ensure training stability and reasonable convergence.

5. In the experiments, why was the peak signal-to-noise ratio (PSNR) = 25 set as the stopping threshold? Why not choose a higher PSNR value?

We appreciate the reviewer’s question. The PSNR (a hyperparameter) = 25 stopping threshold was selected based on empirical observations during test-time optimization. In our ultra-sparse view setup (e.g., 1–5 projection views), we found that PSNR values above 25 already corresponded to visually and structurally meaningful reconstructions. Setting a higher threshold (e.g., PSNR ≥ 30) offered marginal improvements while significantly increasing the computational burden and risk of overfitting to noise or limited view information. Moreover, our goal was not to achieve the maximum PSNR of the model inference to the referenced sparse views, but rather to perform efficient fine-tuning sufficient for realistic anatomical rendering. We found that PSNR ≈ 25 served as a practical and consistent early stopping criterion across different test cases. Nonetheless, we agree that adaptive or dynamic stopping strategies based on perceptual metrics can be explored in future work for deploying TomoGRAF into clinics.

6. During the experimental process, your model's baseline performance was established using a single AP view. To determine model performance, reconstructions were additionally performed with 1, 2, 5, and 10 views. The view angles were specified as follows: for 1-view reconstruction, the AP view was used as reference; for 2-view reconstruction, AP and lateral views were used for inference; for 5-view reconstruction, starting from the AP view, every 72° rotation was applied to cover the full 360°; for 10-view reconstruction, starting from the AP view, every 36° rotation was applied to cover the full 360°. However, in the Discussion section, the statement "Meanwhile, TomoGRAF, besides 1-view referencing, can leverage additional X-ray views at arbitrary angles" appears to lack strong experimental support, since the experimental procedure in this study clearly defined view angles with uniform angular intervals, which seems inconsistent with "arbitrary angles". We recommend either: (1) adding experiments to demonstrate the value of arbitrary angles (e.g., using only the AP view and its adjacent angles) for TomoGRAF during inference, or (2) rephrasing this statement to better align with the actual experimental conditions.

We thank reviewer for pointing this out. We have revised our statement in Discussion to “In addition to single-view referencing, TomoGRAF is capable of incorporating multiple X-ray views from diverse directions, as demonstrated in this study using uniformly distributed acquisition angles.”

7. TomoGRAF requires fine-tuning during the inference phase. For specific fine-tuning, the trained prior model is optimized under the supervision of the patient's 2D sparse view projections to adapt to new patient anatomies. Does this mean that during the inference phase, fine-tuning is required for projection images from every angle used? If so, this would lead to increased inference time, and when more views are used for inference, the time would increase accordingly. The paper mentions that single-view reconstruction takes approximately 344 seconds, while each additional view roughly doubles the reconstruction time, which appears relatively slow for practical applications. Can the network be improved to enhance its computational efficiency?

We appreciate the reviewer’s concern regarding inference efficiency. To clarify, fine-tuning is not performed independently for each projection view. Instead, during test-time optimization, random image patches from all available views are sampled and fed into the model in a unified optimization loop. As the number of input views increases, the model benefits from more diverse supervision, which may slightly increase the number of iterations needed to converge, but this increase is *sublinear* (as demonstrated in Figure 1 in this response letter) rather than linear. In our experiments (Table 2), we observed that while reconstruction time increases with additional views, the marginal cost per view decreases, given improved convergence behavior.

We agree that inference speed is a crucial aspect for practical deployment. Therefore, strategies to improve computational efficiency have been thoroughly discussed in the Limitations section of the original manuscript as “First, TomoGRAF requires further fine-tuning at the inference stage, which increases the reconstruction time (1-view at 344.25±10.32 s and 2-view at 719.46±26.78 s). The time further increases with inference using more views. Significant acceleration is desired for online procedures such as motion adaptive radiotherapy (59). Model compression techniques such as network pruning (60) and quantization (61) can decrease computational complexity while maintaining accuracy. Additionally, hardware acceleration via TensorRT (62) optimization or specialized processors (e.g., FPGAs (63), TPUs (64)) could also potentially improve the inference speed. Architecturally, incorporating efficient neural representations (e.g., lightweight MLPs (65) or hash-based encoding (66)) and adaptive sampling (67) methods could reduce computational overhead by prioritizing critical regions. Future work will explore these optimizations to improve TomoGRAF’s feasibility for real-time clinical applications, which would be essential for interventional procedures.” and the content has been highlighted in green in the manuscript.

**Figure 1**: Relationship between TomoGRAF inference time and number of referenced sparse views.

8. In the quantitative analysis, this paper employed SSIM, PSNR, and RMSE metrics, but it did not thoroughly discuss in the qualitative analysis whether these metrics can reflect clinical diagnostic requirements. In clinical applications, the accuracy of CT images is crucial. Have you considered inviting clinicians to evaluate the generated predicted CT images and assess their feasibility?

We thank the reviewer for highlighting this important consideration. We agree that conventional quantitative metrics such as SSIM, PSNR, and RMSE, while commonly used in the literature, do not fully capture the diagnostic relevance of reconstructed CT images. In this study, our focus was on establishing a technical proof-of-concept for the TomoGRAF framework, and as such, we did not incorporate clinical reader evaluations. That said, we acknowledge the value of involving clinical experts in future evaluations, especially as we move toward applying this framework to real patient data. We added a note in the Discussion section to reflect this important point and outline plans for future clinical validation as “Lastly, while the current study primarily focuses on demonstrating the technical feasibility of ultra-sparse view reconstruction of the proposed TomoGRAF framework, we recognize that conventional quantitative image quality metrics may not fully capture the clinical utility of reconstructed images. As a future direction, incorporating clinical evaluation, such as qualitative scoring by radiologists or task-based diagnostic assessment, will be informative to assess the real-world applicability and reliability of TomoGRAF in clinical practice.”

9. The pseudocode of the Siddon algorithm (Appendix 1) serves as an important methodological supplement. Please further clarify its specific implementation details in TomoGRAF, such as how it integrates with the fully connected network - does the network directly learn attenuation coefficients, or is this achieved through post-processing?

We thank the reviewer for this insightful question. Siddon’s ray tracing algorithm serves as the forward projection operator within the TomoGRAF framework, replacing the original ray tracing in NeRF method for natural lights to compute line integrals through the reconstructed volume.

Specifically, the fully connected network directly outputs voxel-wise attenuation coefficients that represent the 3D volume. Siddon’s algorithm then integrates these coefficients along rays corresponding to the given projection views to produce synthetic 2D projections. This forward projection step is fully differentiable and embedded within the network training loop, allowing end-to-end optimization. There is no separate post-processing step for attenuation coefficients; the network learns to represent the volume implicitly, and Siddon’s algorithm models the physics of X-ray projection during both training and inference stages.
